# Addition of triple negativity of breast cancer as an indicator for germline mutations in predisposing genes increases sensitivity of clinical selection criteria

**DOI:** 10.1186/s12885-018-4821-8

**Published:** 2018-09-26

**Authors:** Juliane Hoyer, Georgia Vasileiou, Steffen Uebe, Marius Wunderle, Cornelia Kraus, Peter A. Fasching, Christian T. Thiel, Arndt Hartmann, Matthias W. Beckmann, Michael P. Lux, André Reis

**Affiliations:** 1 Institute of Human Genetics, Friedrich-Alexander-Universität Erlangen-Nürnberg (FAU), Schwabachanlage 10, 91054 Erlangen, Germany; 2 Department of Gynecology and Obstetrics, University Hospital Erlangen, Comprehensive Cancer Center Erlangen-EMN, Friedrich-Alexander-Universität Erlangen-Nürnberg (FAU), Universitätsstr. 21-23, 91054 Erlangen, Germany; 3 Institute of Pathology, University Hospital Erlangen, Friedrich-Alexander-Universität Erlangen-Nürnberg (FAU), Krankenhausstr. 8-10, 91054 Erlangen, Germany

**Keywords:** Breast cancer, TNBC, Next-generation sequencing, Gene panel, Mutational spectrum

## Abstract

**Background:**

Breast cancer is the most common cancer in women. 12–15% of all tumors are triple-negative breast cancers (TNBC). So far, TNBC has been mainly associated with mutations in BRCA1. The presence of other predisposing genes seems likely since DNA damage repair is a complex process that involves several genes. Therefore we investigated if mutations in other genes are involved in cancer development and whether TNBC is an additional indicator of mutational status besides family history and age of onset.

**Methods:**

We performed a germline panel-based screening of 10 high and low-moderate penetrance breast cancer susceptibility genes (*BRCA1*, *BRCA2*, *ATM*, *CDH1*, *CHEK2*, *NBN*, *PALB2*, *RAD51C*, *RAD51D* and *TP53)* in 229 consecutive individuals affected with TNBC unselected for age, family history or bilateral disease. Within this cohort we compared the number of mutation carriers fulfilling clinical selection criteria with the total number of carriers identified.

**Results:**

Age at diagnosis ranged from 23 to 80 years with an average age of 50.2 years. In 57 women (24.9%) we detected a pathogenic mutation, with a higher frequency (29.7%) in the group manifesting cancer before 60 years. Deleterious *BRCA1* mutations occurred in 14.8% of TNBC patients. These were predominantly recurrent frameshift mutations (24/34, 70.6%). Deleterious *BRCA2* mutations occurred in 5.7% of patients, all but one (c.1813dupA) being unique.

While no mutations were found in *CDH1* and *TP53*, 10 mutations were detected in one of the six other predisposition genes. Remarkably, neither of the *ATM*, *RAD51D*, *CHEK2* and *PALB2* mutation carriers had a family history. Furthermore, patients with non-*BRCA1/2* mutations were not significantly younger than mutation negative women (*p* = 0.3341). Most importantly, among the 57 mutation carriers, ten (17.5%) would be missed using current clinical testing criteria including five (8%) with BRCA1/2 mutations.

**Conclusions:**

In summary, our data confirm and expand previous studies of a high frequency of germline mutations in genes associated with ineffective repair of DNA damage in women with TNBCs. Neither age of onset, contralateral disease nor family history were able to discern all mutation positive individuals. Therefore, TNBC should be considered as an additional criterion for panel based genetic testing.

## Background

Breast cancer is the most common cancer in women. 12–15% of all tumors lack immunohistochemical expression of estrogen receptor (ER), progesterone receptor (PR) and human epidermal growth factor receptor 2 (HER2) [1] and are therefore termed triple-negative breast cancers (TNBC). Patients with TNBC are characterized by a high risk of relapse and poor prognosis [2] and are likely to receive chemotherapy since receptor specific therapies are ineffective. TNBC is more common among African Americans and western sub-Saharan Africans compared with White/Caucasian Americans and Europeans [3]. Besides, TNBC disproportionately affects young women [4].

Overall, women are more likely to be tested for breast/ovarian cancer susceptibility genes if they fulfill certain criteria. According to current guidelines, genetic testing in Germany is recommended in BC patients, who have at least a 10% prior probability of carrying a *BRCA1/2* mutation based on clinical criteria as age of manifestation, family history and contralateral disease [5] and, only since end of 2016, considering receptor triple negativity before age 50. In the years 2007 and 2008, when gene testing was restricted to *BRCA1* and *BRCA2* several studies have demonstrated that *BRCA1*-mutation carriers are more likely to be diagnosed with TNBC than non-carriers [6]. Consequently testing in TNBC patients, if at all performed, was often restricted to *BRCA1* only. However, though TNBCs constitute nearly 80% of *BRCA1*-associated breast cancers [7], *BRCA1* mutations have only been found in a subset of TNBC patients [8]. Therefore a BRCA1/2-centered perspective may ignore the significance of other predisposing genes. Their presence seems likely since DNA damage repair by homologous recombination is a complex process that involves several other genes besides *BRCA1* and *BRCA2*.

Recent technological advances such as high-throughput sequencing technologies have enabled cost-effective simultaneous interrogation of multiple risk genes. Though most familial breast cancer studies grouped all cancer subtypes together, few studies like Couch et al. (2015) evaluated genetic predisposition to a specific subtype like TNBC. The identification of mutation carriers is clinically of increased importance. As management conventions [9–11] are becoming established, more breast cancer patients will request testing to get access to specific therapies, regardless of their age and a priori lower risk. This requires appropriate selection criteria for genetic testing.

Therefore, we now investigated all 229 female individuals with TNBC at a single center over a period of 54 months to assess whether a diagnosis of TNBC is an additional indicator for a germline mutation in breast cancer predisposing genes.

## Methods

All patients with TNBC, newly diagnosed or in aftercare, treated in the Department of Gynecology and Obstetrics between 01/2012 and 06/2016 were referred for diagnostic purposes to our interdisciplinary outpatient clinic.

Clinical data (including a three-generation pedigree) and informed consent for diagnostic testing were collected. All 229 unrelated females received genetic testing. One hundred thirty-eight patients were tested immediately after TNBC diagnosis. Ninety-one patients were in aftercare during the selection process (> 1–20 years after cancer diagnosis). Two hundred twenty-three women were of German origin (97.4%), four from Russia, one from Hungary and one from Thailand. 110 (48%) women had at least one relative with breast cancer. Family history was defined as at least one affected relative regardless of kinship degree. Cancers occurring through two unaffected females at > 60 years of age were discounted.

ER and PR receptor status was determined by immunohistochemistry and classified as negative if less than 1% of cells showed stained nuclei in tumor cells. HER2 status was considered as negative, if IHC scores were 0 and + 1. IHC scores of + 2 were also classified as negative in case of negative fluorescence in situ hybridization results for HER2. FISH was considered positive if the Her2/CEN 17 ratio was > = 2.0 or the majority of tumors cells showed at least 6 Her2 gene signals.

Genomic DNA was extracted according to standard procedures with an automated chemagic MSM I system (Perkin Elmer, Baesweiler, Germany). A targeted resequencing kit, the TruSight Cancer Sequencing Panel, was used for library preparation and sequencing on a MiSeq platform (Illumina, San Diego, CA, USA). All procedures were performed according to the manufacturers’ instructions. Library preparation with the TruSight Rapid Capture was done using 50 ng of genomic DNA per sample. For sequencing the prepared library was applied to MiSeq Flowcell. Paired sequences obtained were mapped to human genome reference GRCh37/hg19 using BWA-MEM version 0.7.7 [12]. Ten genes (*BRCA1/2*, *ATM*, *CHEK2*, *PALB2*, *RAD51C*, *RAD51D*, *NBN*, *CDH1* and *TP53*) were analyzed with the SeqNext module of the Sequence Pilot software (JSI medical systems GmbH, Kippenheim, Germany).

For detection of copy number variants (CNVs), the SeqNext CNV analysis module from the Sequence Pilot software package was used. All coding exons of the analyzed genes served both as control and targets using the analysis mode “all versus all”. Identified CNVs were confirmed by multiplex ligation-dependent probe amplification (MLPA) analyses using the appropriate SALSA MLPA kits (*BRCA1*: P002; *RAD51C*: P260; *CHEK2*: P190) (MRC Holland, Amsterdam, The Netherlands) according to the manufacturer’s instructions.

To predict the potential impact of the identified nonsynonymous germline variants on protein function we used 5 web-based algorithms: UMD-Predictor [13], SIFT [14], Polyphen-2 [15], Mutation Taster [16] and additionally, ALIGN-GVGD [17, 18]. Suspected splice site mutations were tested by three different web-based splicing effect prediction tools, Splice Site Prediction by Neural Network [19], NetGene2 Server [20, 21], and Human Splice Finder (HSF 3.0) algorithm [22] to correlate splicing probabilities for wild type and mutated sequences. Frequencies of variants were compared with European-American and African-American control samples from the Exome Variant Server online database [23, 24]) and with 60,706 unrelated individuals sequenced as part of various disease-specific and population genetic studies from the Exome Aggregation Consortium (ExAC) [25] to exclude rare polymorphisms. All *p*-values were calculated using a Mann-Whitney Rank sum test; boxplots and p-value calculations were done in R version 2.15.3.

## Results

We screened a total of 229 TNBC patients regarding mutations in one of the following breast cancer susceptibility genes: *BRCA1*, *BRCA2*, *ATM*, *CDH1*, *CHEK2*, *NBN*, *PALB2*, *RAD51C*, *RAD51D* and *TP53*. In 57women (24.9%) we detected a pathogenic mutation, with a higher frequency (29.7%) in the group manifesting cancer before 60 years (Table 1).

**Table 1:** Age distribution of patients and their mutation status in relation to family history

	Overall			Family History			No family history		
Age	Number	Mutation	%	Number	Mutation	%	Number	Mutation	%
< 36	40	17	42.5%	28	16	57.2%	12	1	8.3%
36–60	142	37	26%	64	29	45.3%	78	8	10.2%
> 60	47	3	6.3%	10	1	10%	37	2	5.4%
overall	229	57	24.9%	102	46	45%	127	11	8.7%

*BRCA1* (34 cases, 56.6%) followed by *BRCA2* (13 cases, 21.6%) represented the most frequently mutated genes. While no mutations were found in *CDH1* and *TP53*, 10 mutations (17.5%) were detected in one of the 6 other predisposition genes (Table 2, Table 3). No individual presented more than one mutation.

**Table 2:** Mutations in breast cancer risk genes and number of positive diagnostic criteria for gene testing

Mutated gene	Number of diagnostic criteria				
	0	1	2	3	Total
BRCA1	3	18	11	2	34
BRCA2	2	7	3	1	13
CHEK2	1	1	0	0	2
NBN	0	1	0	0	1
RAD51C	0	2	1	0	3
RAD51D	1	0	0	0	1
PALB2	2	0	0	0	2
ATM	1	0	0	0	2
Overall	10	29	15	3	57

Each column delineates the number (0–3) of fulfilled diagnostic criteria for gene testing (0–3) for each of the risk-genes. One point was given each for diagnosis before age 35 years, for bi- or contralateral breast cancer/ ovarian cancer and for family history. Ten women out of 57 (17.5%) did not fulfill any of these criteria and would thus otherwise go untested

**Table 3:** Mutations in BC/OC susceptibility genes identified and clinical presentation in respective patients

#	Exon	cDNA-change	Predicted AA-change	BIC	GnomAD	Age at diagnosis	Contra-lateral disease	Other cancers	Family BC/OC history
	BRCA1								
1	2	c.68_69delAG	p.(Glu23Valfs*17)	185delAG	1.987e^−4^	46	–	34 y lymphoepithelial cancer Repeated skin cancer of unknown origin	BC
2	5	c.181 T > G	p.(Cys61Gly)	C61G	3.255e^−5^	38	–	–	BC
3	5	c.181 T > G	p.(Cys61Gly)	C61G	3.255e^−5^	43	–	36 y/ 42 y basalioma	OC
4	11	c.904delG	p.(Ala302Leufs*12)	1023delG	0	30	–	–	BC, OC
5	11	c.962G > A	p.(Trp321*)	W321X	8.237e^−6^	29	–	–	BC 3rd degree relative
6	11	c.1127delA	p.(Asn376Ilefs*18)	1246delA	0	23/23	yes	–	BC
7	11	c.1396delC	p.(Arg466Glyfs*9)	–	0	45	–	–	BC, OC
8	11	c.1504_1508delTTAAA	p.(Leu502Alafs*2)	1623del5	8.135e^−6^	40/54	yes	–	BC, OC
9	11	c.1504_1508delTTAAA	p.(Leu502Alafs*2)	1623del5	8.135e^−6^	54	–	–	BC, OC
10	11	c.1916 T > A	p.(Leu639*)	L639X	0	34	–	–	BC, OC
11	11	c.1916 T > A	p.(Leu639*)	L639X	0	42	–	–	BC, OC
12	11	c.2411_2412delAG	p.(Gln804Leufs*5)	2530delAG	0	37	–	–	–
13	11	c.2411_2412delAG	p.(Gln804Leufs*5)	2530delAG	0	33	–	–	BC, OC
14	11	c.2411_2412delAG	p.(Gln804Leufs*5)	2530delAG	0	33	–	–	–
15	11	c.2411_2412delAG	p.(Gln804Leufs*5)	2530delAG	0	45/52	yes	–	BC
16	11	c.2411_2412delAG	p.(Gln804Leufs*5)	2530delAG	0	53	–	–	BC
17	11	c.3481_3491del	p.(Glu1161Phefs*3)	3600del11	0	40	–	–	BC, OC
18	11	c.3481_3491del	p.(Glu1161Phefs*3)	3600del11	0	46	–	–	BC, unk. Abd. canc.
19	11	c.3481_3491del	p.(Glu1161Phefs*3)	3600del11	0	65	–	–	–
20	11	c.3627dupA	p.(Glu1210Asnfs*11)	3746ins A	0	59	–	–	BC
21	11	c.3700_3704delGTAAA	p.(Val1234Glnfs*8)	3819del5	0	43/47	yes	–	BC
22	11	c.3700_3704delGTAAA	p.(Val1234Glnfs*8)	3819del5	0	50	–	–	BC, OC
23	11	c.4035delA	p.(Glu1346Lysfs*20)	4154delA	4.066e^−6^	43	–	–	BC
24	11	c.4676-1G > A		IVS15-1G > A	0	30/30	yes	–	BC 4th degree relative
25	13–15	Deletion exon 13–15		–	0	58	–	–	BC
26	20	c.5194_5277del	p.(His1732_Lys1759del)	Exon20del	0	32/36	yes	–	BC
27	20	c.5266dupC	p.(Gln1756Profs*74)	5382insC	0	33	–	–	–
28	20	c.5266dupC	p.(Gln1756Profs*74)	5382insC	0	33	–	–	BC, OC
29	20	c.5266dupC	p.(Gln1756Profs*74)	5382insC	0	30/48	yes	–	–
30	20	c.5266dupC	p.(Gln1756Profs*74)	5382insC	0	35	–	–	BC, unk. Abd. canc.
31	20	c.5266dupC	p.(Gln1756Profs*74)	5382insC	0	39	–	–	BC, unk. Abd. canc.
32	21–24	c.5278-?_5592 +?del	Deletion exon 21–24	–	0	33	–	–	BC, unk. Abd. canc.
33	21	c.5291 T > C	p.(Leu1764Pro)	L1764P	0	42	–	–	BC
34	24	c.5492delC	p.(Pro1831Leufs*3)	6511delC	0	45	–	–	BC
	BRCA2								
1	5	c.516 + 2 T > A		IVS6 + 2 T > A	0	41	–	–	BC
2	10	c.1813dupA	p.(Ile605Argfs*9)	2041ins A	0	29	–	–	BC
3	10	c.1813dupA	p.(Ile605Argfs*9)	2041ins A	0	29	–	–	BC
4	11	c.2244_2245del	p.(Tyr748*)	2472delCA	0	31	–	–	–
5	11	c.5303_5304delTT	p.(Leu1768Argfs*5)	5531delTT	4.075e^−6^	42	–	–	OC
6	11	c.5616_5620delAGTAA	p.(Lys1872Asnfs*2)	5844del5	4.126e^−6^	42	–	–	–
7	11	c.6486_6489del4	p.(Lys2162Asnfs*5)	6714del4	8.785e^−6^	29/33	yes	–	BC 3rd degree relative
8	15	c.7180A > T	p.(Arg2394*)	R2394X	0	51/64	yes	–	BC
9	15	c.7617 + 2 T > G		7829 + 2 T > G	0	53	–	–	BC
10	17	c.7878G > C	p.Trp2626Cys	W2626C	4.064e^−6^	63	–	–	BC
11	20	c.8560delT	p.(Tyr2854Metfs*9)	8788delT	0	38/52	yes	–	BC
12	22	c.8755-1G > A		IVS21-1G > A	0	68	–	–	BC, OC
13	23	c.8992_9025del34	p.(Ser2998Ilefs*19)	–	0	60	–	–	–
#	Exon	cDNA-change	Predicted AA-change	ClinVar (number of reported individuals)	GnomAD frequency	Age at diagnosis	Contra-lateral disease	Other cancers	Family BC/OC history
	RAD51C								
1	7	c.955C > T	p.(Arg319*)	Pathogenic (5)	8.128e^−6^	30	–	–	BC
2	8	c.1026 + 5_ + 7delGTA		Likely pathogenic (5) Pathogenic (1)	0	43	–	64 y non-Hodgkin-lymphoma	BC
3	8	c.1026 + 5_ + 7delGTA		Likely pathogenic (5) Pathogenic (1)	0	51	–	–	BC, unk. Abd. canc.
	RAD51D								
1	7	c.577-2A > G		Likely pathogenic (1)	0	52	–	–	–
	CHEK2								
1	4	c.470 T > C	p.(Ile157Thr)	Likely pathogenic (4) Pathogenic (7) Unknown (3)	5.061e^−3^	50	–	–	BC 3rd degree relative
2	4	c.470 T > C	p.(Ile157Thr)	Likely pathogenic (4) Pathogenic (7) Unknown (3)	5.061e^−3^	47	yes	–	–
	NBN								
1	6	c.657_661del5p	p.(Lys219Asnfs*16)	Pathogenic (12) Risk factor (1)	1.988e^−4^	56	–	66 y OC	BC
	ATM								
1	26	c.3802delG	p.(Val1268Terfs*1)	Pathogenic (5)	3.972e^−5^	40	–	–	–
	PALB2								
1	4	c.1227_1231delTGTTA	p.(Tyr409*)	–	0	44	–	25 y melanoma	BC 3rd degree relative
2	11	c.3165C > A	p.(Tyr1055*)	Pathogenic (1)	0	47	–	–	–

*BC* breast cancer, *OC* ovarian cancer, *unk. Abd. canc* abdominal cancer of unknown origin

Deleterious *BRCA1* mutations in 14.8% of TNBC patients (Table 2, Table 3). These were predominantly frameshift mutations (24/34, 70.6%). The most frequent mutations both among them and in total were the founder mutations c.5266dupC and c.2411_2412delAG. Of the 20 different mutations identified 17 were previously reported as disease causing (International Database of Breast Cancer, https://research.nhgri.nih.gov/bic/), the remaining three comprised large deletions affecting exons 13–15 and 21–24 respectively and the frameshift mutation c.1396delC.

Deleterious *BRCA2* mutations occurred in 5.7% of patients, all but one (c.1813dupA) being unique, including 11 truncating mutations (seven frameshift, three splice mutations and one nonsense) and one missense mutation (c.7878G > C). Despite the frameshift mutation c.8992_9025del34 all other alterations were listed in the International Database of Breast Cancer.

Mutations in non-*BRCA1/2* predisposition genes were identified in 22% of mutation carriers (13/60) (Table 2, Table 3). In total, mutations in *NBN* and *RAD51D* were found in 3 individuals each, mutations in *ATM*, *CHEK2* and *PALB2* in 2 individuals each and one mutation in *RAD51*C (Table 2). Overall, 13 out 229 tested individuals (5.7%) carried a non-*BRCA1/2* mutation.

**Fig. 1:**
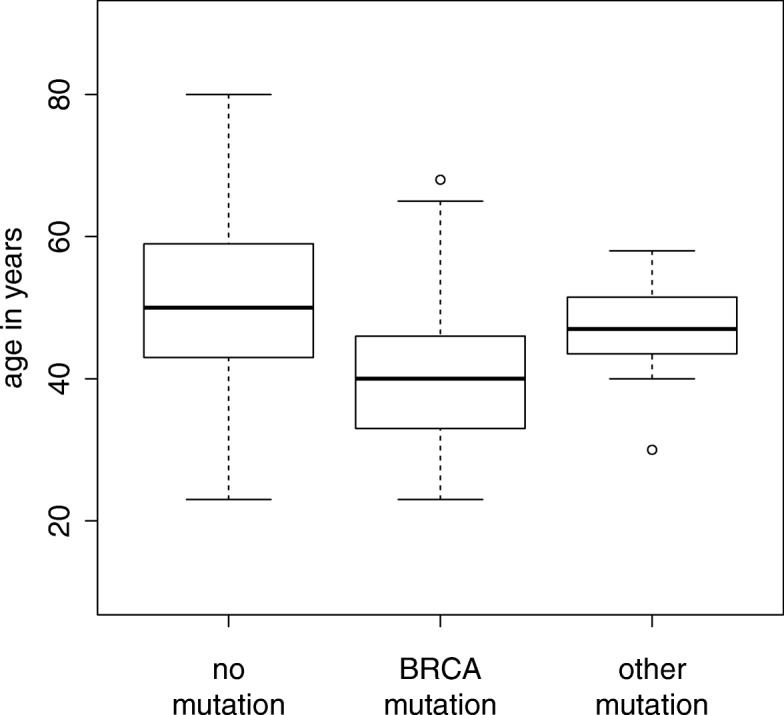
Age at first manifestation in relation to the mutation status. Box-plot diagram of age at first manifestation of breast cancer reveals that women with a BRCA1/2 mutation manifest significantly earlier than those without a mutation or a mutation in one of the other genes

Of the patients examined, 79.5% were younger than 60 years. Only 3 mutation carriers developed cancer beyond 60 years of age, which represents 5% of mutation carriers and 6.3% of their age group. Almost one third of all deleterious mutations (29.8%) were detected in very young women aged 35 years or less (Table 1). The median age at diagnosis was significantly younger for *BRCA1* (40 years) and *BRCA2* (41.5 years) carriers compared to patients without a mutation (50 years, *p* = 2.286e-05; Mann-Whitney) or compared to non-*BRCA1/2* mutation carriers (50 years, *p* = 0.3341) (Fig. 1). In contrast, patients with non-*BRCA1/2* mutations were not significantly younger than mutation negative women (*p* = 0.5288) (Fig. 1).

**Fig. 2:**
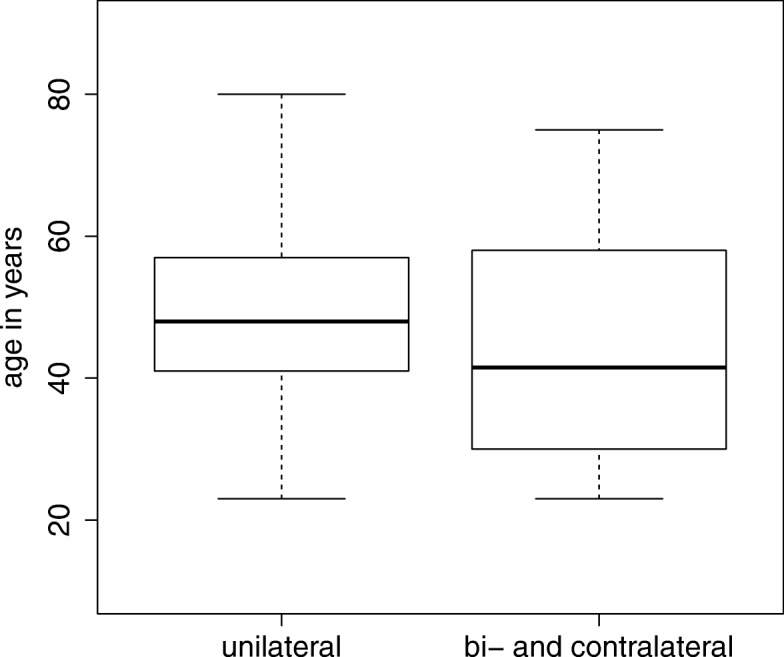
Age at first manifestation in dependence of unilateral or bi- and contralateral manifestation. Box-plot diagram of age at first manifestation of breast cancer reveals that women with bi-or contralateral manifestation (*n* = 18) manifest earlier than those with unilateral disease (*n* = 211)

Eighteen individuals of the entire group developed bi- or contralateral disease at latest 18 years after initial diagnosis. While in the unilateral group only 21.8% (*n* = 46) had a mutation, the majority of the bi- or contralateral affected was mutation positive (*n* = 11; 61.1%) 12 women had a family history (66.7%), but interestingly only nine of them were mutation positive. Overall, women with bi- or contralateral disease developed cancer at significantly younger age with a difference of median age of 8.5 years compared to those with unilateral disease (*p* = 0.01215; Mann-Whitney) (Fig. 2).

**Fig. 3:**
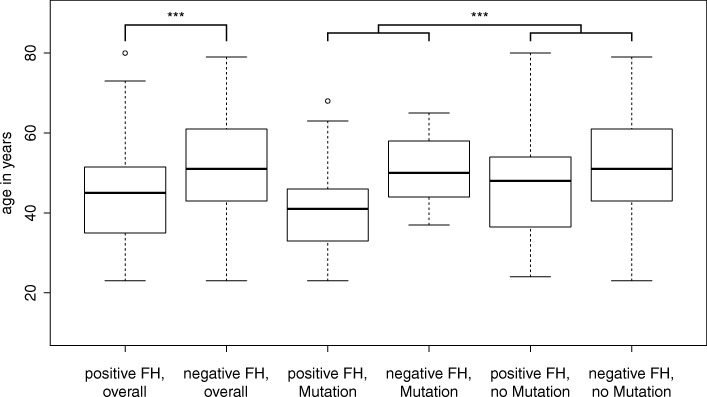
Age at first manifestation in relation to mutation status and family history. Box-plot diagram of age at first manifestation reveals that overall women with a positive family history manifest earlier than those without and that mutation positive women manifest earlier when compared to mutation negative women of their respective family history group

A considerable number of women with TNBC had a family history (44.5%) of whom a mutation was found in 45% (Table 1). Among the *BRCA1/2* mutation carriers, even 86.7% had at least one affected relative. Interestingly, family history had an independent influence on age at diagnosis (Fig. 3). Taken as a whole, women with family history had a median age at diagnosis 6 years earlier than those without (*p* = 0.00057). This difference was lost in mutation carriers (Fig. 3, middle) while it remained in cases without a detected mutation. Remarkably, neither of the *ATM*, *RAD51D*, *CHEK2* and *PALB2* mutation carriers had a family history, when considering first and second degree family members.

## Discussion

We identified a germline mutation in 24.9% of 229 TNBC patients. 17.5% of individuals were diagnosed before age 36 and 79.4% before the age of 60 (Table 1). As reported previously, the mutation detection rate decreased with higher age at diagnosis (Sharma et al., 2014). More than 95% of patients with a germline mutation were diagnosed with breast cancer before age 60. Among the 47 cases with age > 60, only three (6.3%) were mutation positive.

Overall, we found a significant earlier disease manifestation in those women who have a family history (mean 45 vs. 51 years; p = 0.00057) (Fig. 3, left). Also 29% of women (37/127) without family history were aged > 60 years as compared to only 9.8% (10/102) of women with a family history (Table 1). Interestingly, this difference is lost in cases with a detected mutation (Fig. 3, middle) while it remains in those without a mutation in any of the investigated genes (Fig. 3 right). This is in agreement with the hypothesis that other genetic factors at single or multiple loci segregating in mutation-negative families predispose to BC/OC [26, 27]. In the mutation-positive individuals the penetrance of the identified mutation apparently overrides this background effect.

Regarding the mutational spectrum, *BRCA1* (34 cases, 56.6%) followed by *BRCA2* (13 cases, 21.6%) represented the most frequently mutated genes. The biological reason for the *BRCA1* preponderance remains unclear, given that both genes collaborate in the homology-directed DNA repair pathway, but BRCA1 may exert a particular role in hormone receptor expression [28].

Most *BRCA1*/2 mutations were truncating, while only four individuals carried a missense mutation (Table 2 and Table 3). By comprehensively investigating genomic alterations using MLPA we found a deletion in an additional 1.3%. Altogether, we identified mutations in 20.5% of individuals, a higher incidence than the 14.6% reported by the largest study to date [29], but in line with other German studies reporting mutations in 21% [30] and 17.5% [28] respectively. In all above mentioned studies large genomic deletions were not investigated. Differences in ancestral background and the use of different genotyping methods may have further contributed to deviating BRCA1/2 mutation frequencies. Nevertheless, in all studies the prevalence of BRCA mutations exceeded 10%, a number often used as an a priori probability threshold in current testing guidelines.

The incidence of mutations varies widely among different populations; some present a wide spectrum of different mutations, while in some particular ethnic groups specific mutations show a higher frequency probably due to founder effects [31]. Three *BRCA1* mutations identified in 34 women (Table 3) were particularly frequent indicating such founder effects. Mutation c.3481_3491del, although found in many geographical areas, is common in France where it accounts for up to 37% of all *BRCA1/2* mutations [32]. The relatively high frequency in our study group may be related to the arrival of large numbers of French Huguenot refugees in the area in the late seventeenth century. Similarly, a second mutation, c.2411_2412delAG, is much more frequent in our study group than in patients from all across Germany [5]). Finally, the mutation c.5266dupC is a known founder mutation and not only the most common *BRCA1* alteration in the German population [5] but also frequently found in multiple, apparently diverse populations [33]. In contrast, for *BRCA2* all but one mutation (c.1813dupA) were unique, in agreement with previous studies [5].

DNA damage repair involves interactions of several specific proteins to restore genomic integrity. Therefore we extended our study also to the respective genes associated with low-moderate penetrance (Table 2) and identified a deleterious germline mutation in six out of eight genes in 10 women (4.3%). From these, one variant (*CHEK2* p.(Ile157Thr) is currently classified as mutation, although we would suggest to reclassify it as a low penetrance risk factor due to its high frequency in large variant databases. This variant is described as associated with BC [34, 35], was found in 0.5% of Europeans in GnomAD [36, 37] (Table 3).

Arguably, *CHEK2* is one of the most important breast cancer susceptibility genes after BRCA1/*BRCA2*. However, besides the variant c.470 T > C no other CHEK2 alteration was identified, suggesting that variants in this gene are not frequently associated with TNBC. Accordingly, on the basis of a study of 47 early-onset *CHEK2*-positive patients, it was concluded that *CHEK2*-positive patients are equally likely to be estrogen receptor (ER) positive as are patients with nonhereditary disease (Cibulsky et al., 2009). The same is true regarding CDH1, associated with lobular BC and gastric cancer [38, 39].

Besides *CHEK2*, mutations were identified in NBN, RAD51C, *RAD51D*, *PALB2* and *ATM.* Mutations in *RAD51D* were mainly associated with ovarian cancer. However, it was suggested that mutations in *RAD51D* could confer a risk of TNBC [40]. PALB2 is a BRCA2-interacting protein that is crucial for key *BRCA2* genome caretaker functions. Nevertheless, recent studies have shown that *PALB2* also interacts with *BRCA1* [41, 42]. Overrepresentation of triple negative status in *PALB2*-related breast cancers was suggested in studies performed in European cohorts [43, 44].

Not unexpectedly for low-moderate penetrance genes, the majority of carriers had no cancer family history. Median age at disease onset was also comparable to that of women without a mutation (Fig. 1). While 91% of women with a *BRCA1/2* mutation show additional clinical signs associated with positive mutation status e.g. age of onset < 50 years, contralateral disease or a positive family history, none of the women with a *RAD51D*, *PALB2* or *ATM* mutation showed any of these signs (Table 2). They would thus have been escaped genetic testing when relying only on criteria developed for high-penetrance genes such as *BRCA1/2*. When grouping patients by criteria used for assessing test eligibility, 10 of the women aged > 35 did not fulfil any of them (Table 2). Thus neither age of onset < 50 years, contralateral disease nor family history are able to discern all mutation positive individuals, suggesting that TNBC should be considered as an independent criterion for genetic testing.

Besides family history, age at diagnosis and mutations in specific genes, hormone receptor status is considered as a risk factor of developing a new primary breast cancer in the contralateral breast [45, 46]. In our study, 18 women had bi- or contralateral disease in the further course. Of these, 10 women carried a BRCA1/2 mutation and one woman the *CHEK2* variant p.(Ile157Thr). Thus, 11 (61.1%) were mutation positive, a much higher frequency when compared to 46/211 (21.8%) women with unilateral disease. On the other hand, in the mutation-negative fraction only 7 out of 169 (4.15%) patients developed a second breast cancer indicating a small risk after excluding mutations in the known genes. Women with bi- or contralateral disease were significantly younger (median 8.5 years) when compared to those with unilateral disease (Fig. 2). Of the 6 women without a mutation, 3 had a family history, suggesting an underlying genetic predisposition. It is possible that a mutation outside the coding area or in a gene not covered by this investigation is predisposing in these individuals.

## Conclusion

In summary, our data confirm and expand previous studies of a high frequency of germline mutations in genes known to be associated with ineffective repair of DNA damage by homologous recombination in women with TNBCs. Many of these mutations would be missed using current restrictive testing criteria. Therefore, gene panel based mutation testing should be offered to all women diagnosed with TNBC, irrespective of age or family history.

Furthermore, genetic testing has become a compelling predictive tool, as advanced targeted therapeutic agents, such as poly(ADP-ribose) polymerase (PARP) inhibitors emerge, that selectively induce synthetic lethality in tumor cells deficient in homologous recombination repair [47]. Identifying mutations in genes associated with homologous recombination may expand the number of tumors eligible for PARP inhibitors. Furthermore, presence of such mutations may allow dosage reduction of chemotherapeutic agents e.g. platinum treatment, thereby minimizing the risk of associated severe hematologic toxicities [48]. Moreover, once the disease causing mutation is identified, predictive testing can also be offered to all adult family members and where appropriate, risk-reducing preventive medical interventions [49].
